# *Cystoisospora suis* in Portugal: an observational study of prevalence, management, and risk factors

**DOI:** 10.1186/s40813-023-00328-8

**Published:** 2023-07-12

**Authors:** Tiago Nunes, Vassilis Skampardonis, Francisco Costa, Maria Antónia da Conceição, Daniel Sperling

**Affiliations:** 1 CEVA Saúde Animal, R. Dr. António Loureiro Borges 9/9A 9ºA, 1495-131 Algés, Portugal; 2 Laboratory of Epidemiology, Biostatistics and Animal Health Economics, University of Thessaly, 224 Trikalon St, 43100 Karditsa, Greece; 3 Polytechnic of Coimbra, Study Center on Natural Resources, Environment and Society (CERNAS), Coimbra Agriculture School (ESAC), 3045 Coimbra, Portugal; 4 CEVA Santé Animale, 10 Avenue de la Ballastière, 33500 Libourne, France

**Keywords:** *Cystoisospora suis*, Coccidia, Portugal, Piglet, Toltrazuril, Disinfection

## Abstract

**Background:**

Neonatal coccidiosis is a common and important disease of suckling piglets in modern farming caused by *Cystoisospora suis*. Prevalence rates are high, namely, in Portugal, although no recent data are available. The metaphylactic administration of a single dose of toltrazuril and hygienic measures are the backbone of control strategies on positive farms. However, several studies have shown that these programs are not always effective, underlining the need to revise the risk factors and control strategies currently applied. The present study evaluated *C. suis* prevalence on Portuguese farms and assessed the risk factors associated with facilities and farm rearing practices.

**Results:**

From the 27 tested farms, 23 were positive to *Cystoisopora suis* (85.2%). In total, 258 litters were sampled (accounting for 516 samples—2 samples per litter), with an average of 59.7% of positive litters per positive farm. Faecal pools from litters, in which liquid faeces predominated had a higher probability of containing oocysts than litter pools with mainly solid (Odds Ratio: 9.87; *p* < 0.0001) or pasty faeces (OR: 7.05; *p* = 0.001), and samples obtained from younger animals had higher oocyst counts (coefficient: − 0.0720; 95% CI − 0.125; − 0.019). No significant effect of toltrazuril administration was observed on the positivity rate, and none of the tested farms used disinfectants with official claims against parasites and known anticoccidial effects (e.g., cresol-based products).

**Conclusions:**

The *C. suis* prevalence on Portuguese swine farms appears to be similar to the prevalence found in other European countries. Repeated sampling of the same litter and the use of autofluorescence microscopy after a modified Ritchie technique seems to have increased sensitivity and consequently the detection rate of positive litters. Finally, despite the common use of oral toltrazuril, *C. suis* control programs appear to not always be effective (based on the detection of oocysts in faecal samples), suggesting the need to revise the control strategies applied in the field, including management factors and choices of disinfectant products.

## Background

*Cystoisospora suis* is the causative agent of neonatal coccidiosis in piglets and is the most critical protozoan that causes disease in swine, with high prevalence rates reported in swine-producing countries, including Portugal [1–3].

Contaminated farrowing crates are the main source of infection for naïve suckling piglets, and after the initial infection, typically, not all animals of the same litter or of different litters in the same herd are affected equally, and different degrees of disease severity are observed [1, 4]. Usually, after a prepatent period of 4 to 6 days, piglets develop pasty to watery nonhemorrhagic diarrhoea, and the most heavily affected animals show a reduction in growth or even weight loss [2]. The age at infection of the animals is negatively correlated with the severity of the clinical signs and oocyst excretion, with younger animals presenting greater oocyst shedding and clinical signs [5, 6]. Shedding animals present a biphasic pattern of oocyst excretion [2], with peaks that do not necessarily coincide with the presence of diarrhoea, which complicates parasite diagnosis [4]. Although pasty faeces are suggested to be more likely positive and contain higher levels of oocysts [1], the correlation between oocyst excretion and faecal scoring is rather problematic, and variable reports are available [7, 8].

The diagnosis is based on the detection of oocysts in faeces and is routinely carried out using different flotation protocols combined with conventional microscopy techniques [2]. However, this technique presents some constraints, namely, a low detection threshold (333 oocysts per gram of faeces, OPG). Therefore, by presenting a higher sensitivity, autofluorescence microscopy is recommended and is frequently used for diagnosis and prevalence studies [8, 9].

Control of neonatal coccidiosis in piglets relies upon the early metaphylactic treatment of infected piglets with anticoccidials, such as toltrazuril, and cleaning procedures of the farrowing room [1, 2]. Applying a single-dose treatment with oral toltrazuril (3–5 days of age, DOA) reduces the probability and severity of oocyst shedding, as well as the occurrence of diarrhoea, and is becoming a generalized practice among swine farms [10–12]. Nevertheless, observational studies based on the assessment of oocysts in the faeces of piglets showed that the control of neonatal coccidiosis is not always effective, even in toltrazuril-using farms, probably due to poor timing or quality of application by oral drenching [13, 14]. Considering that the farrowing pen environment is the main source of infection, the proper cleaning and disinfection of the facilities between farrowing batches also plays a key role in infection control. Removing faecal matter and reducing the relative humidity of the facilities was proven to reduce the oocyst load and viability [15], which is usually achieved at farm level by establishing a drying period after cleaning. The duration of drying period necessary to effectively reduce the oocyst load is variable depending on weather and farm conditions. In addition, disinfection after cleaning reduces the pressure of infection more effectively if performed with disinfectants that have known anticoccidial action, such as cresol-based products [16, 17].

This observational study aimed to evaluate the prevalence of *C. suis* on intensive pig production farms in Portugal through the detection of parasite oocysts in piglet faeces combined with the analysis of risk factors associated with facilities and rearing practices.

## Results

### *Cystoisospora suis* prevalence rate

In this study, 258 litters from 27 different swine farms were sampled. To overcome the wave pattern of excretion, each litter was sampled twice, 24 to 48 h prior to weaning and approximately one week before. The average age for 2nd sampling varied between 15.4 and 26.1 days of life, with the first sampling taking place between the 8.4 and 22.1 days of life in average.

Of the 27 tested farms, 23 were identified as positive for oocyst excretion, accounting for a positivity rate of 85.2% (considering a positive farm whenever at least one oocyst was detected in one litter). On positive farms, within-farm prevalence showed an average of 59.7% litters, ranging between 18.2 and 100% (Table 1).

**Table 1:** Number of sampled litters per farm (N = 27), positivity rate (%), average age (days) per sampling occasion, range of oocyst count and average oocyst count per positive sample

Farm	Global			First sampling			Second sampling			Oocyst count range*
	Number of litters	Positive litters	%	Age (days)	Positive litters	%	Age (days)	Positive litters	%	
ACA	9	7	77.8	15.7	6	66.7	22.7	6	66.7	1–31(14.2)
AGA	10	8	80.0	16.3	8	80.0	23.3	4	40.0	1–23 (6.9)
ALA	12	0	0.0	11.2	0	0.0	18.2	0	0.0	–
EUA	10	9	90.0	13.8	7	70.0	20.8	8	80.0	1–289 (45.3)
EUB	10	2	20.0	8.4	2	20.0	15.4	0	0.0	1- 1 (1.0)
IGA	8	5	62.5	17.1	1	12.5	24.1	5	62.5	1–5 (2.0)
IGB	8	8	100.0	14.3	8	100.0	20.3	7	87.5	2–127 (18.2)
IGC	13	0	0.0	18.0	0	0.0	23.2	0	0.0	-
IGD	10	8	80.0	22.1	7	70.0	28.1	2	20.0	1–8 (2.2)
ISA	10	3	30.0	14.3	3	30.0	21.3	2	20.0	2–86 (20.0)
ISB	10	6	60.0	9.8	3	30.0	20.8	6	60.0	1–62 (19.8)
MJA	10	6	60.0	13.8	4	40.0	18.8	4	40.0	1–16 (6.1)
MQA	11	2	18.2	18.4	1	9.1	22.7	1	9.1	3–11 (7.0)
MQB	8	0	0.0	19.8	0	0.0	24.8	0	0.0	-
PCA	10	7	70.0	14.8	4	40.0	21.8	4	40.0	1–6 (2.5)
RCA	10	3	30.0	9.9	2	20.0	23.9	3	30.0	1–63 (25.8)
RPA	9	7	77.8	15.1	6	66.7	22.1	4	44.4	1–35 (7.3)
RPB	10	4	40.0	11.6	2	20.0	17.6	4	40.0	1–56 (12.8)
SFA	10	5	50.0	22.0	2	20.0	26.0	4	40.0	4–57 (16.3)
SPA	9	7	77.8	11.4	5	55.6	21.4	5	55.6	1–59 (11.0)
STA	10	2	20.0	18.6	1	10.0	25.6	2	20.0	1–42 (15.0)
SVA	10	4	40.0	10.8	3	30.0	17.8	2	20.0	1–1 (1.0)
VSA	8	6	75.0	20.1	4	50.0	26.1	5	62.5	1–13 (4.0)
VSB	6	0	0.0	16.7	0	0.0	22.7	0	0.0	-
VSC	9	8	88.9	19.0	8	88.9	25.0	6	66.7	1–36 (7.4)
VSD	8	2	25.0	14.8	1	12.5	20.8	2	25.0	1–23 (8.67)
VSE	10	10	100.0	14.3	10	100.0	19.3	8	80.0	1–104 (16.8)

*Oocyst count per sample, considering only the positive samples. Between brackets the average count per farm

### Identification of statistically significant variables

During the screening phase for the detection of eligible variables (significant at the 0.25 level) for inclusion in a full model, the following risk factors were identified:

- For part I of the two-part model (the logistic part), the use of toltrazuril, the age of piglets in sampled litters, litter size and faecal consistency were qualified.
- For part II of the two-part model (linear part), the use of toltrazuril, number of collected samples, production type of the farm, number of workers occupied in the farrowing rooms, farrowing room drying period, age of piglets in sampled litters, faecal consistency and herd size were qualified.

The aforementioned variables were those included in the full two-part model, which was further reduced by backward elimination until variables that were significant at the 0.05 level remained. After selection of the parsimonious multivariable model, none of the tested interaction terms were significant (*p* > 0.05), while the farrowing room drying period was identified as significant and retained in the final model during the forward selection process. The final two-part model included one factor in the logistic part and two factors in the linear part, namely, faecal consistency, age of piglets in sampled litters and farrowing room drying period, respectively.

### Age at sampling

**Fig. 1:**
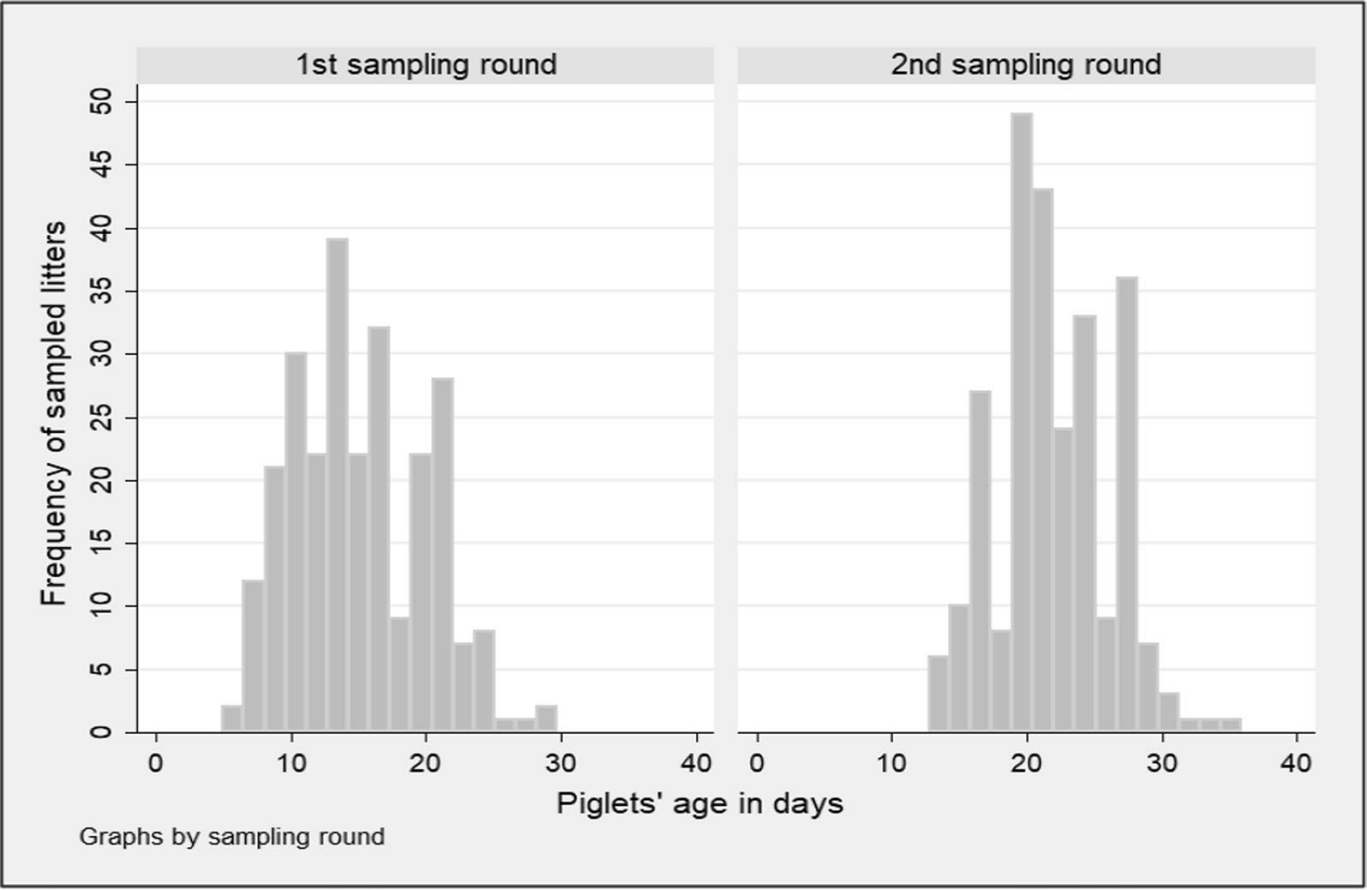
Distribution of sampled piglets’ age by sampling round, expressed as relative frequencies

**Fig. 2:**
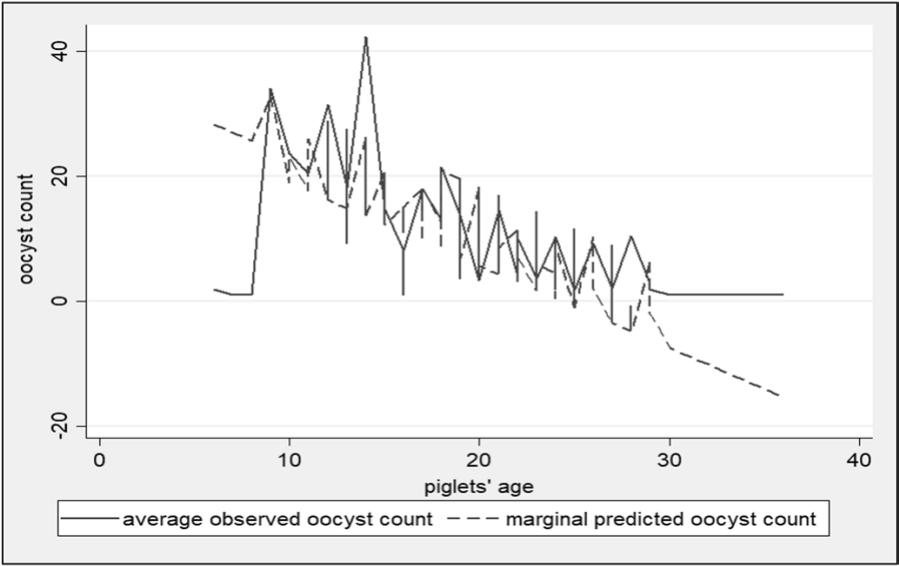
Average observed versus model predicted marginal oocyst counts over piglets’ age, conditional on occurrence of oocyst excretion

The timepoint used to schedule the sample collection was the weaning age: each litter was sampled 24 to 48 h prior to weaning and the week before. Because the weaning age varied among farms, the age at sampling varied to the same degree (Fig. 1). The first sample was taken at 15.3 days of life on average (median: 14.8 days; range: 8–22 days). The second sample was collected on average at 22.0 days of life (median: 22.1 days; range: 15–28 days). Although no significant association was found between the age at sampling and the probability of oocyst shedding (p = 0.06), there was a significant association between the age at sampling and oocyst count (*p* = 0.00178), with higher counts in samples from younger animals (coefficient: − 0.0720; 95% Confidence Interval: − 0.125; − 0.019) (Fig. 2).

### Faecal consistency score (FS) and oocyst excretion

Overall, 60.9% of the faecal samples were pasty (FS = 2), and 11.0% were liquid (FS = 3). The remaining 28.1% were well formed, solid faeces (FS = 1). On the first sampling, 81.4% (n = 210) of the litters had an FS > 1, but only 38.0% (n = 98) were positive for oocysts. On the second sampling, the percentage of litters with FS > 1 diminished to 62.4% (n = 161), but the percentage of oocyst-shedding litters remained similar (36.4%; n = 94).

**Fig. 3:**
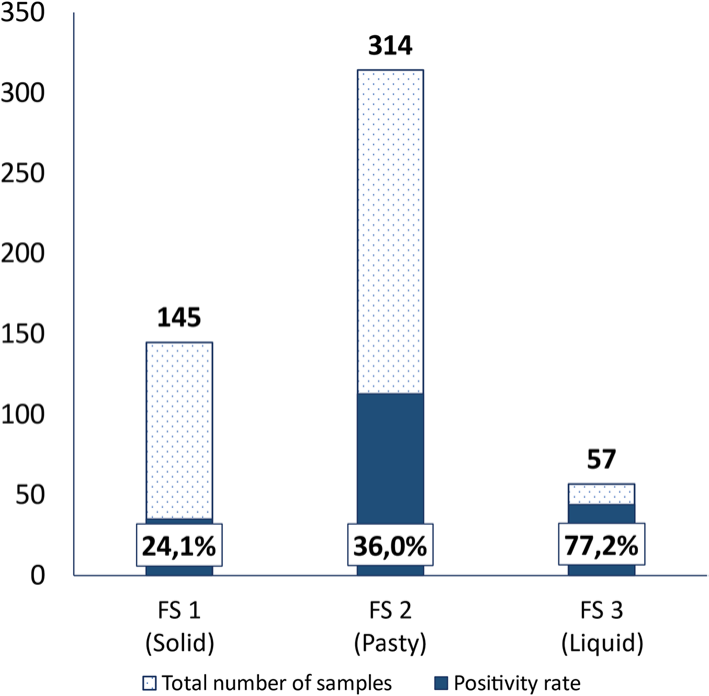
Distribution of samples and positivity rate (%) per faecal consistency score (FS)

Oocysts were found for all FSs, with 24.1% (35/145) of positive samples with FS = 1, 36.0% (113/314) with FS = 2, and 77.2% (44/57) with FS = 3 (Fig. 3). The average oocyst count on FS-1 samples was 3.32 oocysts per sample, with higher counts on FS-2 and FS-3 samples (5.14 and 8.91 respectively).

Despite the presence of oocysts in all FS grades, a higher FS was associated with the probability of oocyst excretion (*p* = 0.0006). In litters with liquid faeces, the probability of oocyst excretion was 9.87 times higher than that in litters with solid faeces (*p* < 0.001) and 7.05 times higher than that in litters with pasty faeces (*p* = 0.001). No difference in the odds of excretion was found between litters with solid and pasty faeces (*p* = 0.271).

Another important observation was the association between FS and age (*p* = 0.001): older piglets were less likely to have higher faecal consistency scores (OR: 0.907, 95% Confidence Interval: 0.857; 0.960); specifically, for one unit increase of age it was 1.10 times (95% Confidence Interval: 1.04; 1.167) less likely to have higher versus lower FS (score 3 & 2 versus 1, or score 3 vs 2 & 1).

### Risk factor assessment

On each farm, a written survey was completed to evaluate the risk factors. The farm managers answered the questions on a voluntary basis (Table 2):

- *Production system and sow herd size*: 70.4% of the farms (19/27) were farrow-to-finish units, and the remaining 29.6% (8/27) were piglet production units. The average herd size was 624.8 sows per farm, with 55.6% of the herds (15/27) having between 301 and 600 sows, 25.9% of herds (7/27) having between 601 and 1000 sows and the remaining 18.5% of the herds (5/27) having more than 1000 sows.
- *Clinical history of neonatal diarrhoea and application of anticoccidial substances*: 92.6% (25/27) of farms had a clinical history of preweaning diarrhoea in the last 2 years. Of the 27 tested farms, 20 (74.0%) were using oral toltrazuril regularly, even though 12 of them reported a neonatal diarrhoea history and 18 applied toltrazuril within the first 3 days of the piglets’ life. The remaining 7 farms were not using any anticoccidial substance. No significant effects were observed on the positivity of the litters (*p* = 0.762).
- *Number of workers in the farrowing room and cross-fostering strategy*: 70.4% of the farms had 2 workers fully dedicated to the farrowing unit, while 22.2% had only 1 worker. The remaining 7.4% of farms had more than 2 workers. The cross-fostering strategy of the farms varied among them: 44.4% of the farms reported cross-fostering piglets only in the first 3 days of life, and the other 44.4% reported cross-fostering piglets even after the third day of life. The remaining 11.1% of farms reported cross-fostering only on the first day of the piglets’ life. No significant effect of the cross-fostering strategy or the number of workers was found on *C. suis* positivity (*p* = 0.841 and *p* = 0.185, respectively).
- *Animal flow, cleaning, and disinfection strategies*: 77.8% of the farms had an *all-in/all-out* strategy in the farrowing units, while the remaining 22.2% had a continuous flow of animals. A total of 81.5% of the farms reported using detergent when cleaning the farrowing rooms between batches, with the remaining 18.5% using only water to clean the facilities. None of the farms applied a disinfectant with a known anticoccidial effect after cleaning the farrowing rooms. No significant effect of animal flow or biosecurity strategies on *C. suis* positivity was found (*p* = 0.979 and *p* = 0.512, respectively). Regarding the room drying period (time window between batches while the farrowing rooms are kept empty and drying after cleaning and disinfection), 77.8% of the farms reported having a drying period of 24 h or less. The remaining farms declared that they kept rooms empty for 48 h (7.4%) or more (14.8%). A significant effect of the drying period was found on the oocyst count (*p* = 0.0005), with higher oocyst counts in samples from farms with a drying period of more than 48 h: 25.55 oocysts per sample in average versus 12.15 oocysts in 48 h drying period, 11.40 oocysts in 24 h drying period and 8.98 oocysts in < 24 h drying period.

**Table 2:** Risk factors present per farm

Farm	*C. suis* positivity	Production type	Herd size	Nb workers	Uses toltrazuril	Toltrazuril until 3rd day	Neonatal Diarrhea History	Cross-fostering time	*All in/ All out*	Drying period	Uses detergent	Anticoccidial disinfectant
ACA	Yes	FF	360	2	Yes	Yes	Yes	24 h	yes	48 h	No	No
AGA	Yes	FF	420	1	No	No	Yes	> 72 h	no	< 24 h	Yes	No
ALA	**No**	FF	600	2	Yes	Yes	Yes	> 72 h	yes	24 h	Yes	No
EUA	Yes	FF	620	2	No	No	Yes	72 h	yes	> 48 h	No	No
EUB	Yes	FF	800	2	No	No	Yes	72 h	yes	24 h	Yes	No
IGA	Yes	PP	1100	2	Yes	Yes	Yes	> 72 h	yes	< 24 h	Yes	No
IGB	Yes	FF	900	2	Yes	Yes	Yes	> 72 h	yes	< 24 h	Yes	No
IGC	**No**	PP	1200	2	Yes	Yes	Yes	> 72 h	yes	< 24 h	Yes	No
IGD	Yes	FF	320	2	Yes	Yes	Yes	24 h	yes	24 h	No	No
ISA	Yes	FF	610	2	Yes	Yes	Yes	> 72 h	yes	24 h	Yes	No
ISB	Yes	FF	340	2	No	No	Yes	72 h	yes	> 48 h	Yes	No
MJA	Yes	FF	600	1	Yes	Yes	Yes	72 h	yes	24 h	Yes	No
MQA	Yes	PP	1000	2	Yes	Yes	Yes	72 h	yes	> 48 h	Yes	No
MQB	**No**	FF	350	2	Yes	Yes	Yes	72 h	yes	< 24 h	Yes	No
PCA	Yes	PP	320	1	No	No	Yes	72 h	yes	24 h	Yes	No
RCA	Yes	FF	640	2	Yes	Yes	Yes	24 h	yes	24 h	Yes	No
RPA	Yes	PP	310	2	Yes	Yes	No	72 h	yes	24 h	Yes	No
RPB	Yes	FF	600	1	Yes	Yes	No	72 h	yes	48 h	Yes	No
SFA	Yes	FF	650	2	Yes	No	Yes	> 72 h	yes	24 h	No	No
SPA	Yes	FF	1200	2	Yes	Yes	Yes	72 h	no	< 24 h	Yes	No
STA	Yes	FF	500	2	No	No	Yes	72 h	yes	> 48 h	No	No
SVA	Yes	FF	1200	5	No	No	Yes	72 h	no	< 24 h	Yes	No
VSA	Yes	PP	350	2	Yes	No	Yes	> 72 h	yes	< 24 h	Yes	No
VSB	**No**	PP	700	3	Yes	Yes	Yes	> 72 h	no	< 24 h	Yes	No
VSC	Yes	PP	380	2	Yes	Yes	Yes	> 72 h	yes	< 24 h	Yes	No
VSD	Yes	FF	400	1	Yes	Yes	Yes	> 72 h	no	< 24 h	Yes	No
VSE	Yes	FF	400	1	Yes	Yes	Yes	> 72 h	no	< 24 h	Yes	No

Farms with 100% negative samples are shown in bold

FF: farrow-to-finish farm; PP: piglet production farm. Herd size: number of sows per farm; Nb of workers: number of workers fully dedicated to the farrowing unit. Cross fostering time: time limit to cross-foster piglets after birth

## Discussion

This cross-sectional study aimed to assess the prevalence of neonatal porcine coccidiosis and its associated factors in commercial (industrial-scale) piglet-producing farms in Portugal. In this study, we performed sampling in the regions with the highest pig production that represent approximately 90% of Portugal’s sow herd [18]. The positivity rate for *C. suis* found in this study (85.2%) was higher than the values previously described for Portuguese farms (64%) and neighbouring countries, such as Spain (40%) and France (56%) [3]. However, it is crucial to consider the higher sensitivity and lower detection limit of the laboratory technique used (AF) in the current study [8], which may account for the observed increase in the positivity rate. Simultaneously, the repeated sampling performed in the present study contributed to overcoming the biphasic nature of oocyst shedding, as 16.3% of the litters were confirmed to be positive only at the second sampling. Since individual patent periods (shedding) can be very short, piglets in a litter become infected at different time points, and repeated and multiple sampling is necessary to detect *C. suis* on a positive farm and should be proposed as a standard sampling strategy [8, 11]. A more recently published paper that used a similar methodology (AF) found similar positivity rates on farms from Spain (83.3%) [13]. The within-farm positivity rate of 59.7% found in the present study is also aligned with the results presented by the same authors for other European countries: an average 50.1% positivity rate (ranging between 11.5% on Austrian farms and 78.2% on Spanish farms) [13].

The age at sampling had a significant effect on oocyst excretion and general susceptibility to cystoisosporosis, with age-related susceptibility described in the literature [5, 6]. The age range of the piglets at the first sampling occasion (8.4–22.1 days) coincided with the peak prevalence described by Hamadejova and Vitovec [7]. Moreover, the age range of the piglets at the second sampling occasion (15.4–28.1 days) coincided with the downward prevalence phase described by the same authors. Although the endogenous development of *C. suis* is among the fastest of the coccidia, the prepatent period is commonly 5 days, whereas *Eimeria* species usually require 7–10 days for their endogenous development [19]. From that perspective, sampling during the first week of age is not considered a standard procedure. Therefore, the effect of piglet age on the oocyst rate and litter positivity, although noticeably clear in the present study, applies only to a specific time window in the piglets’ life and cannot be extrapolated to animals under 7 days of age.

Although diarrhoea is the main symptom of coccidiosis, the relationship between faecal consistency score (FS) and oocyst shedding is unclear, with different authors describing different relationships between FS and oocyst detection. In the present study, the effect of FS was significant, with liquid faeces having a higher probability of containing oocysts, as in previous reports [7]. In contrast, some previous studies reported [13] that oocysts were significantly more often observed in pasty faeces, which can be attributed to the fact that semiliquid and liquid faecal matter is difficult to collect when samples are taken from slatted or perforated floors, so diarrheic faeces might have been underrepresented in the sampling, although diarrhoea was present. It is also important to take in consideration that some published works compare faecal score and oocyst shedding in individual animals along time, and not pooled samples from litters, as in the present study, which may contribute to the observed differences.

In this study, a significant association was observed between FS and sampling age, with liquid faeces coming tendentially from younger animals and solid faeces coming from older piglets. Since the age of sampling has a relevant effect on the positivity of the samples, with younger animals presenting higher positivity rates [5, 6], it cannot be excluded that the outcome for FS might have been influenced by the age of the animals, with FS-3 having higher positivity due to the lower average-age of the donors.

Furthermore, younger animals are more prone to develop coinfections (neonatal diarrhoea) caused by several pathogens besides *C. suis*, such as ETEC strains of *Escherichia coli*, *Clostridium perfringens* type A*,* or rotavirus, among others. In our study, 92.6% of the tested farms had a history of preweaning diarrhoea in the last 2 years. The objective of our study was not to assess mixed infections and other causative agents of diarrhoea; consequently, the FS (liquid faeces representing diarrhoea) could not be fully attributed only to *C. suis,* despite the high detection rate of oocysts in diarrheic samples. On the other hand, *C. suis* infections are known to induce intestinal microbiota disruption [20, 21], exacerbating the severity and duration of the original symptoms, and the synergistic effect of *C. suis* with other enteropathogens has been described. It is possible that the litters with liquid faeces in this study were in a more advanced stage of infection, with established intestinal dysbiosis (hence progressing to liquid faeces) and closer to the peak of oocyst excretion (for most infected piglets, the peak occurs after the onset of diarrhoea) [1, 2, 7]. More detailed and controlled studies are needed to better understand the relationship between FS and oocyst excretion and to provide clear guidelines for practitioners on what type of samples should be collected to diagnose *C. suis* infection. The current recommendation, which should be followed in the field, reflecting the previously discussed relationship of FS and oocyst shedding, is to collect faecal samples of different consistencies (in similar proportions when possible) to increase the chance of detecting *C. suis* infection.

Risk factors were assessed based on voluntarily completed written surveys, with no posterior validation of the provided information. In addition, the farm managers answered based on the rearing protocols established for each farm rather than on the effective farm practices, which may diverge. Therefore, the obtained results need to be interpreted with caution.

The results showed the generalization of several practices among Portuguese farms: most farms used oral toltrazuril on a regular basis (74.0%) and detergent to clean the facilities (81.5%) but had a short drying period after disinfection (24 h or less on 77.8% of farms), and none of the farms used disinfectants with confirmed anticoccidial effects. The high positivity rate for *C. suis* even on toltrazuril-using farms corroborates the findings of other authors [13, 14]. Furthermore, it underpins the need for an in-depth review of coccidia control at the farm level (including the route, dose and timing of toltrazuril administration, in addition to cleaning and disinfection strategies).

A longer drying period is expected to create an environment with lower humidity, a highly adverse condition to oocyst viability, thus reducing the pressure of infection. Under experimental conditions, the viability of the oocysts was reduced to zero after 24 h with relative humidity under 62% and temperatures over 25 °C [15]. However, in the present study, the samples from farms with longer drying periods had no significant effect on positivity rate. These samples had even higher oocyst counts per sample, which seems contradictory to the established knowledge. Nevertheless, these results must be analysed considering the following:

- The average oocyst count on farms with longer drying periods was influenced by two farms with very high average oocyst counts. These farms were not using detergent to clean the facilities, raising some doubts about the effectiveness of the cleaning procedures.
- The drying period was used as a proxy to assess the reduction in humidity after disinfection (the real risk factor), but no direct assessment of the humidity of the facilities was carried out.
- The selected time periods (less than 24 h, 24 h, 48 h, over 48 h) might have been too short to obtain an adequate reduction in humidity under field conditions together with differences based on the heating systems used on farms.

Studies with longer time intervals and a less harmonized set of samples may provide clearer data on this topic.

## Conclusions

The results showed a positivity rate of 85.2% for *Cystoisospora suis* in Portugal, which is aligned with the results obtained in recent years in neighbouring countries. The farm positivity of *C. suis* in our study was higher than that previously described by Torres [3] (64.0%), probably due to the higher sensitivity of the sampling strategy (repeated sampling) and the laboratory technique used (autofluorescence microscopy after a modified Ritchie technique) [8]. The faecal consistency scores of the collected samples and the age of the sampled animals had a significant effect on the presence of oocysts, with liquid faeces and samples from younger animals being more frequently positive and with a higher load of detected oocysts. These observations suggest that pooled samples including liquid faeces from piglets with clinical diarrhoea in the second and third weeks of life should be part of the sampling strategy to increase the chance of correctly diagnosing parasite infestation. Furthermore, despite the observed frequent use of oral toltrazuril to prevent coccidiosis, the control of *C. suis* appears to not be sufficiently effective on most farms, suggesting the need to review the strategies used, including treatment protocols, and cleaning and disinfection practices. A vital change would be the proper use of disinfectants with known anticoccidial effects.

## Methods

### Sample and data collection

From 27 Portuguese industrial swine farms (> 100 sows, exclusive indoor production) selected by convenience of sampling according to the farmer’s willingness to participate, 516 pooled samples of piglet faeces were collected from 08/10/2020 to 14/04/2022. The farm manager of each farm filled out a written survey to collect data on the risk factors and provided informed consent to participate in this study.

The sampled litters were selected by convenience sampling and proportionally to the parity order distribution of the corresponding farrowing batch. From 17 farms, faeces were collected from 10 litters per farm. On the remaining 10 farms, where less than 10 litters were available, the totality of available litters was sampled (8.2 litters on average), according to the following criteria: (i) each pool corresponded to one litter only; (ii) the samples were collected at the weaning age on each farm (24 to 48 h before weaning) and one week before that (every litter was sampled twice, resulting in 516 samples from 258 litters in total); (iii) when litters with diarrhoea were present on the farm, at least one of the pools came from one of those litters; and (iv) the consistency of the faeces was assessed during the sample collection and assigned a faecal score of 1 (solid and formed faeces), faecal score of 2 (pasty faeces) or faecal score of 3 (liquid faeces). The faecal score was assigned to each pool according to the most representative faecal consistency present in the sample and samples from all consistencies were included in the study.

All farms were allowed to keep their usual rearing practices during the study, including the weaning age. Since the weaning age varied between 16 and 36 days of life, both sampling occasions comprised a wide range of ages.

Fresh faecal samples were collected from a minimum of five different points within the farrowing crate, anticipating that piglets have a natural tendency to use separate locations for elimination with high frequency at a young age [22, 23].

### Oocyst detection

A modified Ritchie technique [24] was used for *C. suis* oocyst detection. Samples of suckling piglets’ faeces (2 g) were suspended in 10 times their volume of tape water and thoroughly mixed with the addition of 12 glass beads. Sample homogenization was achieved with a vortex for 1 min. Then, the suspension was strained through a sieve with 2 layers of gauze (placed over the sieve and funnel), into a 15 ml centrifuge tube, and centrifuged for 10 min at 336 g. The supernatant was discarded and 5 ml of tap water were added and stirred. The mixture was allowed to stand for 10 min, then 10 ml ether was added and shaken vigorously. Finally, the mixture was centrifuged for 10 min, at 336 g. Four layers were then formed, and the bottom one was reserved, and the others discarded. The sediment was observed between slide and coverslip, and the total number of oocysts, in six randomly fields (at 200×magnification) was recorded for each sample. The oocysts detection was carried out with a fluorescence microscope, Nikon Eclipse Ci-L (Nikon, Minato, Japan), under excitation wavelength of 340–380 ηm and emission within the blue light spectrum (about 450 ηm) [9].

### Sample size estimation

Based on the results of previous relevant and similar studies [25], a minimum difference of 35% was expected in the proportion of positive litters between those that received metaphylactic anticoccidial treatment with a toltrazuril compound and those that received no such treatment. Based on the standard sampling formulae, the minimum required sample size for comparing two such proportions equalled 30 litters, 15 for each group. Sample size calculations were performed using Win Episcope [26], assuming a 95% confidence interval and 80% power. However, standard individual-based sample size formulae do not account for between-herd variation [27] when clustering is present. Therefore, estimated sample sizes must be inflated by the variance inflation factor: VIF = 1 + ICC*(ms-1) [28], where ICC is the intraherd correlation coefficient, and ms is the mean number of litters sampled in each herd. Based on previously published data, an ICC for oocyst excretion of 0.4 was adopted, and an ms = 10 litters (according to the study protocol) was assumed, resulting in a VIF equal to 4.6. Thus, a minimum total of 30*4.6 = 138 litters was needed.

### Effect of treatment and various potential risk factors on the odds and level of oocyst excretion

Oocyst counts were semicontinuous data characterized by a large portion of zeros and skewing to the right of the distribution of the nonzero values [10, 25, 29, 30]. Therefore, we employed a two-part model to estimate the effect of treatment on the repeated measurements on oocyst detection and count. In the first part, the model used logistic regression to quantify the probability of oocyst excretion, while in the second part, linear regression was used to model the mean amount of the decimal logarithm of the nonzero oocyst count values [31]. Random effects were incorporated into the model to account for the within-herd and within-litter correlation of observations, allowing simultaneous capture of the biologically plausible fact that higher odds of excretion may also have higher average oocyst counts. This was achieved by the adopted structure, which adjusted for the cross-equation correlation (ρCEC) of the random effects for the same level (herd or litter) between the logistic and linear parts of the model [29, 32]. Finally, sampling weights were used to adjust for the unequal selection probabilities of litters originating from herds of unequal size. Specifically, these weights were denoted as the inverse of the probability that each litter from each herd was selected for inclusion due to the employed sampling design, thus assigning higher weights to litters of larger herds.

All candidate variables were initially screened one by one for model building using a bivariable approach [33]. Toltrazuril treatment was forced into both parts of all models because it is known to reduce both the odds and the level of excreted oocysts [10]. In past years, prophylactic toltrazuril treatment was inherent to the control of swine cystoisosporosis in Portugal. Therefore, any assessment of the potential effect of the candidate variables on the risk and level of oocyst excretion should account for the use of toltrazuril treatment. During this screening phase, a significance level of 0.25 was used [34]. Then, variables with *p* < 0.25 in both or either part were simultaneously entered into a full model, which was subsequently reduced by backwards elimination [35] until only significant (*p* < 0.05) variables remained. When pairs of highly correlated variables were encountered, the selection of the variable to be included in the model was based on biological plausibility. Two-factor interactions were created between the remaining variables and entered one at a time into the model. Finally, a stepwise forward selection process was performed by introducing previously excluded variables to the final model one at a time, ensuring that any of these variables dropped during the backward elimination process but that could add significantly to the final model were included.

## Data Availability

The dataset analysed during the current study is available from the corresponding author on request.

## References

[1] Lindsay DS, Dubey JP, Santín-Duran M. Coccidia and other Protozoa. In Zimmerman JJ, Karriker LA, Ramirez A, Schwartz KJ, Stevenson GW, Zhang (Eds) J. Diseases of Swine. 11th ed. New Jersey: John Willey and Sons, Inc; 2019. p. 1015–1027.

[2] Joachim A, Schwarz L. Coccidia in Swine: Eimeria Species, *Cystoisospora* (sin. *Isospora*) *suis*. In Mehlhorn (eds) H. Encyclopedia of Parasitology. Heidelberg: Springer Berlin; 2015. p. 1–5.

[3] Torres A. Prevalence survey of *Isospora suis* in twelve European countries. In Proceedings of the 18th IPVS Congress; 2004; Hamburg: International Pig Veterinary Society. p. 243.

[4] Sotiraki S, Roepstorff A, Nielsen J, Maddox-hyttel C, Enoe C, Boes J (2008). Population dynamics and intra-litter transmission patterns of *Isospora suis* in suckling piglets under farm conditions. Parasitology.

[5] Mundt H, Joachim A, Daugschies A, Zimmermann M (2003). Population biology studies on *Isospora suis* in piglets. Parasitol Res.

[6] Worliczek H, Mundt H, Ruttkowski B, Joachim A (2009). Age, not infection dose, determines the outcome of *Isospora suis* infections in suckling piglets. Parasitol Res.

[7] Hamadejova K, Vitovec J (2005). Ocurrence of the coccidium *Isospora suis* in piglets. Veterinární Medicína.

[8] Joachim A, Ruttkowski B, Sperling D (2018). Detection of *Cystoisospora suis* in faeces od suckling piglets - When and how? A comparison of methods. Porcine Health Manag J.

[9] Daugschies A, Bialek R, Joachim A, Mundt H (2001). Autofluorescence microscopy for the detection of nematode eggs and protozoa, in particular *Isospora suis*, in swine faeces. Parasitol Res.

[10] Skampardonis V, Sotiraki S, Koustolas P, Leontides L (2010). Effect of toltrazuril treatment in nursing piglets naturally infected with *Isospora suis*. Vet Parasitol.

[11] Mundt H, Mundt-Wustenberg S, Daugschies A, Joachim A (2007). Efficacy of various anticoccidials against experimental porcine neonatal isosporosis. Parasitol Res.

[12] Joachim A, Guerra N, Hinney B, Hodzic A, Karembe H, Shrestha A (2019). Efficacy of injectable toltrazuril-iron combination product and oral toltrazuril against early experimental infection of suckling piglets with *Cystoisospora suis*. Parasit Vectors.

[13] Hinney B, Cvetkovic V, Espigares D, Vanhara J, Waehner C, Ruttkowski B (2020). *Cystoisospora suis* control in Europe is not allways effective. Front Vet Sci.

[14] Pettersson E, Hestad S, Mottus I, Skioldebrand E, Wallgren P (2019). Rotavirus and *Cystoisospora suis* in piglets during the suckling and early post weaning period, in systems with solid floors and age segregated rearing. Porcine Health Manag.

[15] Lankjaer M, Roepstorff A (2008). Survival of *Isospora suis* oocysts under controlled environmental conditions. Vet Parasitol.

[16] Daugschies A, Bangoura B, Lendner M (2013). Inactivation of exougenous endoparasite stages by chemical disinfectants: current state and perspectives. Parasitol Res.

[17] Straberg E, Daugschies A (2007). Control of piglet coccidiosis by chemical disinfection with a cresol-based product (Neopredisan 135–1®). Parasitol Res.

[18] IP INdE. Recenseamento Agrícola—Análise dos principais resultados - 2019. Lisboa; 2021.

[19] Joachim A, Shrestha A. Coccidiosis in pigs. In: P D. Coccidiosis in Livestock, Poultry, Companion Animals, and Humans. Boca Raton: CRC Press; 2020. p. 125–45.

[20] Shrestha A, Metzler-Zebeli BU, Karembe H, Sperling D, Koger S, Joachim A (2020). Shifts in the fecal microbial community of *Cystoisospora suis* infected piglets in response to toltrazuril. Front Microbiol.

[21] Mengel H, Krüger M, Krüger M, Westphal B, Swidsinski A, Schwarz S (2012). Necrotic enteritis due to simultaneous infection with *Isospora suis* and clostridia in newborn piglets and its prevention by early treatment with toltrazuril. Parasitol Res.

[22] Fraser A, Broom D (1997). Farm animal behaviour and welfare.

[23] Sperling D, Calveyra J, Karembe H, Costa E (2022). *Cystoisospora*
*suis* infection in suckling piglets in Brazil: Prevalence and associated factors. Vet Parasitol Reg Stud Rep.

[24] Ritchie LS (1948). An ether sedimentation technique for routine stool examinations. Bulletin of US Army Med Dep.

[25] Skampardonis V, Sotiraki S, Kostoulas P, Leontides L (2012). Factors associated with ocurrence and level of *Isospora suis* oocyst excretion in nursing piglets of Greek farrowe-to-finish herds. BMV Vet Res.

[26] Thrusfield M, Ortega C, Blas Id, Noordhuizen J, Frankena K (2001). Win episcope 2.0: improved epidemiological software for veterinary medicine. Vet Rec.

[27] Donner A, Donald A (1988). The statistical analysis of multiple binary measurments. J Clin Epidemiol.

[28] McDermott J, Schukken Y, Shoukri M (2012). Study design and analytic methods for data collected from clusters of animals. BMC Vet Res.

[29] Liu L, Ma J, Johnson B (2008). A multi-level two-part random effects model, with application to an alcohol-dependence study. Stat Med.

[30] Xie H, McHugo G, Sengupta A, Drake RCR (2004). A method for analyzing longitudinal outcomes with many zeros. Ment Health Serv Res.

[31] Duan N, Manning W, Morris C, Newhouse J (1983). A comparison of alternative models for the demand for medical care. J Bus Econ Stat.

[32] Tooze A, Grunwald G, Jones R (2002). Analysis of repeated measures data with clumping at zero. Stat Methods Med Res.

[33] Martin W. A structured approach fpor analysing survey data and making useful casual inferences. In: Eightth International Symposium on Veterinary Epideniology and Economics; 1997; Paris. p. 31–32.

[34] Mickey R, Greenland S (1987). A study of the impact of cofounder-selection criteria on effect-estimation. Am J Epidemiol.

[35] Hosmer D, Lemeshow S. Applied logistic regression Chichester: Wiley; 1989.

